# The effect of cognitive training on domains of attention in older adults with mild cognitive impairment and mild dementia: A meta-analysis of randomised controlled trials

**DOI:** 10.7189/jogh.13.04078

**Published:** 2023-06-30

**Authors:** Chien-Mei Sung, Hsiu-Ju Jen, Doresses Liu, Christina Yeni Kustanti, Hsin Chu, Ruey Chen, Hui-Chen Lin, Ching-Yi Chang, Kuei-Ru Chou

**Affiliations:** 1 School of Nursing, College of Nursing, Taipei Medical University, Taipei, Taiwan; 2 Department of Nursing, Fu Jen Catholic University Hospital, Fu Jen Catholic University, New Taipei City, Taiwan; 3 Department of Nursing, Taipei Medical University-Shuang Ho Hospital, New Taipei City, Taiwan; 4 Department of Nursing, Wan Fang Hospital, Taipei Medical University, Taipei, Taiwan; 5 Research Center in Nursing Clinical Practice, Wan Fang Hospital Taipei Medical University, Taipei, Taiwan; 6 Sekolah Tinggi Ilmu Kesehatan Bethesda Yakkum Yogyakarta, Indonesia; 7 Department of Neurology, Tri-Service General Hospital, National Defense Medical Center, Taipei, Taiwan; 8 Institute of Aerospace and Undersea Medicine, School of Medicine, National Defense Medical Center, Taipei, Taiwan; 9 Post-Baccalaureate Program in Nursing, College of Nursing, Taipei Medical University, Taipei, Taiwan; 10 Psychiatric Research Center, Taipei Medical University Hospital, Taipei, Taiwan; 11 Neuroscience Research Center, Taipei Medical University, Taipei, Taiwan

## Abstract

**Background:**

Attention is essential to daily life and cognitive functioning, and attention deficits can affect daily functional and social behaviour, such as falls, risky driving, and accidental injuries. However, attention function is important yet easily overlooked in older adults with mild cognitive impairment, and evidence is limited. We aimed to explore the pooled effect of cognitive training on domains of attention in older adults with mild cognitive impairment and mild dementia using a meta-analysis of randomised controlled trials.

**Methods:**

We searched PubMed, Embase, Scopus, Web of Science, CINAHL, PsycINFO, and Cochrane Library for randomised controlled trials (RCTs) up to 3 November 2022. We included participants aged ≥50 years diagnosed with cognitive impairment, with various cognitive training interventions as the intervention measures. The primary outcome was overall attention and the secondary outcomes were attention in different domains and global cognitive function. We calculated the Hedges’ g and confidence intervals (CIs) using a random-effects model to evaluate the effect size of the outcome measures and evaluated heterogeneity using the χ^2^ test and *I^2^* value.

**Results:**

We included 17 RCTs and found that cognitive training interventions improve overall attention (Hedges’ g = 0.41; 95% CI = 0.13, 0.70), selective attention (Hedges’ g = 0.37; 95% CI = 0.19, 0.55), divided attention (Hedges’ g = 0.38; 95% CI = 0.03, 0.72), and global cognitive function (Hedges’ g = 0.30; 95% CI = 0.02, 0.58) in older adults with mild cognitive impairment, but with relatively low effectiveness.

**Conclusions:**

Cognitive training intervention can improve some attention functions in older adults with mild cognitive impairment. Attention function training should also be incorporated into routine activities and long-term sustainability planning to delay the deterioration of attention function in older adults. Besides reducing their risk of abnormal events in daily life (such as falls), it can also improve their quality of life and help reduce the progression of cognitive impairment, achieving early detection of secondary prevention.

**Registration:**

PROSPERO (CRD42022385211).

Population aging is a key global issue today. Besides visible changes in the aging process, the cognitive neural functions of elderly individuals have been targeted as needing attention. Multiple aspects of their abilities decline, including neural structure (gray and white matter), functionality, neurotransmitter reduction, processing speed, attention, memory, and visual-spatial abilities. This affects not only their daily life abilities, but also potentiates cognitive decline-related problems, and in severe cases, mild cognitive impairment, pre-dementia, and dementia, indirectly affecting their families and increasing social burdens [1].

Mild cognitive impairment (MCI) is a transitional stage between normal aging and dementia, characterised by a decline in cognitive function. Approximately 10% to 15% of individuals diagnosed with MCI progress to dementia annually [2]. Working memory, attention, and executive functions are among the cognitive domains that deteriorate earliest in individuals with MCI. The human cognitive system involves complex processes, among which attention is a fundamental component of cognitive processes. Its function is to efficiently adjust and allocate cognitive resources, assist in target selection and concentration of cognitive operations, and provide response degree to relevant stimuli, playing a crucial role in daily tasks and cognitive operations [3]. Attention can be regarded as the basis of cognitive function activity, and it declines with aging. Attention deficit may affect other cognitive functions, and in turn affect daily life functions and social behaviour, such as falling, dangerous driving, and accidental injuries. Previous studies found cognitive training is effective for various aspects of cognitive function in elderly individuals with mild cognitive impairment, such as attention and executive function training [4-8]. Improvements in attention can be achieved by using cognitive training to intervene in activating the prefrontal cortex, effectively increasing its cognitive functions [9]. However, attention function is important for elderly individuals with mild cognitive impairment and is easily overlooked. Attention research has multiple categories (such as sustained attention, selective attention, divided attention, and attentional shifting) and various measurement methods. However, previous integrated analysis studies have focused primarily on individuals with brain damage or attention deficit hyperactivity disorder (ADHD) rather than elderly individuals with mild cognitive impairment or mild dementia [10-12]. Additionally, there is a lack of indicators for overall attention and other categories of attention. Evidence regarding the effects of attention in elderly individuals with cognitive impairment is limited. We aimed to explore the pooled effect of cognitive training on domains of attention in older adults with mild cognitive impairment and mild dementia using a meta-analysis of randomised controlled trials.

## METHODS

### Reporting standard

We used the Cochrane Collaboration's recommendations to design the protocol for the systematic review and meta-analysis [13] and registered it in the Prospective Registered Systematic Reviews (PROSPERO) database (CRD42022385211). We reported this systematic review and meta-analysis in line with the Preferred Reporting Items for Systematic Reviews and Meta-Analyses (PRISMA) 2020 reporting checklist [14].

### Data sources and search strategy

We searched PubMed, Embase, Scopus, Web of Science, CINAHL, PsycINFO, and Cochrane Library from inception to 3 November 2022, to examine the effect of cognitive training on attention in older adults with mild cognitive impairment. We combined medical subject heading (MeSH) terms, keywords, and Boolean operators (AND and OR) develop the search methods, without constraints on language and publication date. Using the population, intervention, comparison, outcomes and study design (PICOS) framework, we used the following search terms (see Table S1 in the **Online Supplementary Document** for detailed search strategy:

− Population (mild cognitive impairment and mild dementia)
− Intervention (received all types of cognitive training intervention)
− Comparison (did not receive cognitive training intervention)
− Outcomes (different domains of attention)
− Study design (“randomized controlled trial”)

We also searched the reference lists of relevant studies and used Google Scholar to identify other potential studies.

### Study selection

We based our inclusion criteria on the PICOS framework to formulate the research question:

− Participants: aged ≥50 years old, diagnosed with mild cognitive impairment or mild dementia, and presenting with symptoms of cognitive dysfunction.
− Intervention: cognitive training interventions, including various types of cognitive training interventions (e.g. cognitive game training, computer-based cognitive training, attention training, etc.)
− Comparison: active control, passive control, or conventional treatment.
− Outcomes: the primary outcome is attention, and the secondary outcomes include attention in different domains and overall cognitive function.
− Study design: randomised controlled trials.

We excluded duplicate studies, studies that did not involve the relevant study population, study protocols, review articles, case reports, conference or poster abstracts, systematic literature reviews or meta-analyses, and letters, and studies that are irrelevant to the topic. After deduplication, two researchers (CMS and CKY) independently searched and screened the titles and abstracts of retrieved articles, followed by full texts for eligibility based on previously listed inclusion and exclusion criteria. We resolved disagreements through discussion among team members.

### Data extraction

Two researchers (CMS and CYK) independently extracted the data on the characteristics of the studies and participants, including author names, publication year, sample size, age, gender percentage, inclusion criteria for cognitive function, intervention measures and frequency, measurement results, measurement tools, and measurement time points. Discrepancies were discussed with a third researcher

### Quality assessment of included studies

We used the Cochrane Risk of Bias Tool, 2nd version (RoB 2.0) [15] to assess the quality of the included studies. The assessment of the risk of bias concentrates on internal validity, i.e. the degree to which the study is free of bias. It is unrelated to external validity (generalisability or applicability) and precision (the extent to which study results are devoid of random error) as part of internal validity. Certain aspects of trial conduct, such as obtaining ethical approval or calculating sample size, are not explicitly related to risk of bias. The key domains evaluated were bias arising from the randomisation process, bias due to deviations from the intended intervention, bias due to missing outcome data, bias in the measurement of the outcome, and bias in the selection of the reported result. We categorised the assessment results as “low risk of bias”, “some concerns”, and “high risk of bias”. We performed an overall assessment to present all the bias results. Two reviewers (CMS and CYK) independently assessed the included studies and assigned quality scores through consensus, resolving disagreements through discussion with a third expert (Table S3 in the **Online Supplementary Document**).

### Statistical methods

We used the Comprehensive Meta-Analysis Software 3.0 (CMA 3.0) [16] for the data analysis. We calculated the effect sizes of each study and integrated them into a standardised common comparison unit called “effect size”, which we estimated using Hedges' g and the confidence intervals (CIs) to predict the inter-group difference in pre- and post-intervention changes, calculated as a weighted standard deviation. Hedges' g is a standard effect size measure commonly used in experimental and quantitative research. It is unaffected by sample size, enabling easier evaluation and interpretation effects [13,17,18]. We interpreted the pooled effect size of 0.2-0.49 as a small effect, 0.5-0.79 as a medium effect, and ≥0.8 as a large effect, with *P* < 0.05 indicating statistical significance [19]. We used Cochrane Q and *I^2^* tests to assess heterogeneity. The Cochrane Q test determines if there is heterogeneity between studies, indicated by a *P*-value of <0.1 [13,20,21]. The *I^2^* value reveals the magnitude of heterogeneity between studies; a larger number indicates greater heterogeneity, quantified as low, medium, and high, with *I^2^* limits of 25%, 50%, and 75%, respectively. If heterogeneity among studies is found, a random-effects model is used for analysis. A funnel plot is based on Egger's intercept test and used to visualise the plot symmetry. Additionally, Egger's intercept test is used to assess publication bias to detect evidence in small studies, where *P* < 0.1 indicates statistically significant publication bias. Finally, the overall results are presented using a forest plot, showing the combined results and 95% CIs.

### Ethical approval

Our study did not require ethical approval, as we used secondary data from previously published studies which obtained informed consent from participants.

## RESULTS

### Study selection

**Figure 1:**
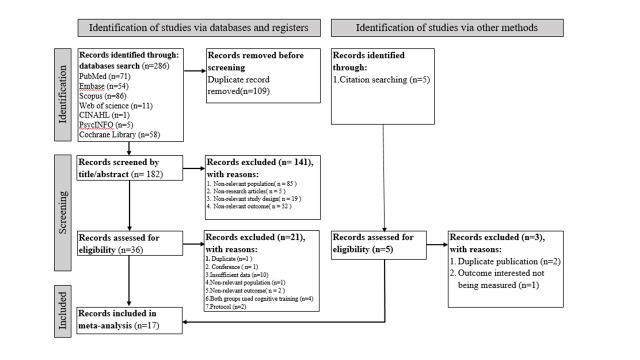
PRISMA flowchart diagram.

We retrieved 286 relevant articles from the databases and located five more by searching the references of the retrieved literature. We imported the references into EndNote 20 (Clarivate Analytics, London, UK) and used its internal deduplication function to remove 109 duplicate articles. We excluded 141 articles after title and abstract screening based on pre-defined inclusion and exclusion criteria and an additional 24 articles after reviewing the full text. We included 17 articles in the meta-analysis **(****Figure 1****).**

### Study characteristics

We included 1128 participants (mean age 69.9 years, 53.13% females) from the randomised controlled trials, which enrolled 16 to 160 participants each. Among the 17 randomised controlled trials [7,8,22-36], four analysed overall cognitive function indicators, ten analysed overall attention, seven analysed selective attention, and three analysed divided attention (Table S4 in the **Online Supplementary Document**).

### Effectiveness on overall attention

**Figure 2:**
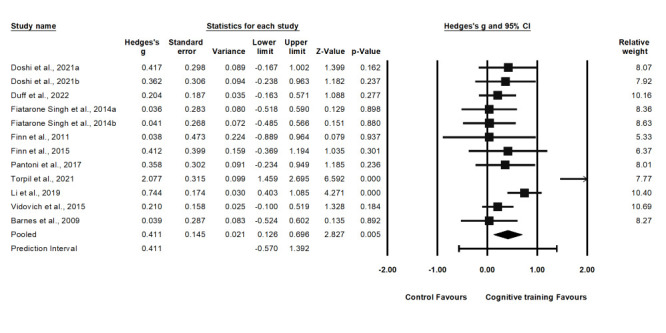
Effect on selective attention.

We included 12 studies on overall attention in the analysis. The pooled Hedge's g was 0.41 (95% CI = 0.13, 0.70) and high heterogeneity (Q-statistic = 40.42, *I^2^* = 72.79%; *P* < 0.001) (**Figure 2**). The pooled effect size from the sensitivity analysis was 0.40 (95% CI = 0.12, 0.69) by removing one study at a time, indicating no significant difference from the primary pooled effect size. Further testing for publication bias showed that the funnel plot visual assessment appeared to be roughly symmetrical (Figure S1 and Table S5 in the **Online Supplementary Document**) The Egger’s regression test also identified no publication bias (*P* = 0.823).

### Effect on selective attention

**Figure 3:**
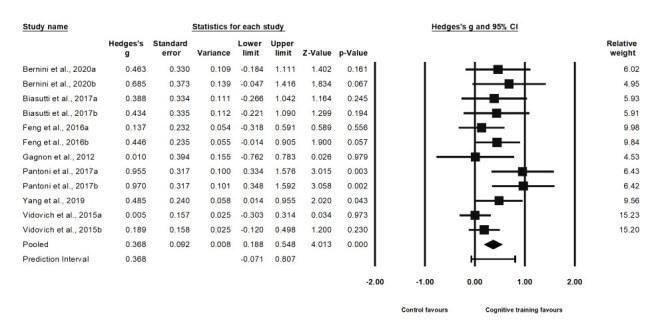
Effect on divided attention.

Twelve studies examined selective attention. The pooled Hedge's g was 0.37 (95% CI = 0.19, 0.55), and we identified low heterogeneity (Q-statistic = 16.21, *I^2^* = 32.13%; *P* = 0.134), with a prediction interval (PrI) of -0.07 to 0.81 **(****Figure 3****)**. The pooled Hedges’s g remained similar following the sensitivity analysis, with an effect size of 0.37 (95% CI = 0.19, 0.55) showing no significant difference from the primary value. Egger's regression test yielded a value of 0.024, indicating the presence of publication bias; we carried out Duval and Tweedie's trim and fill test while considering the missing data to the left of the mean from the funnel plot. The resulting adjusted values showed an effect size of 0.18 by adding five studies and a 95% CI ranging from 0.05 to 0.31, indicating no significant difference from the primary value of the pooled effect size.

### Effect on divided attention

**Figure 4:**
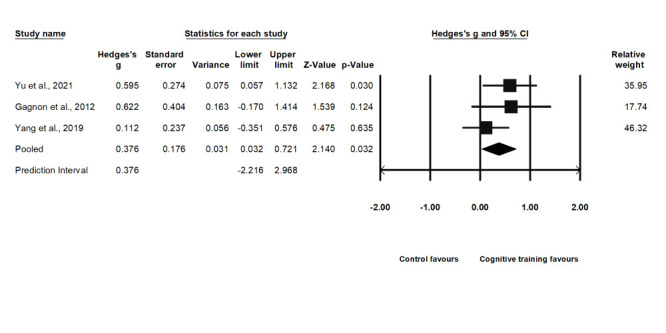
Effect on global cognitive function.

Three included studies examined divided attention. The pooled Hedge's g was 0.38 (95% CI = 0.03, 0.72); we observed low heterogeneity (Q-statistic = 2.25, *I^2^* = 10.97%; *P* = 0.325) and a PrI of -2.22 to 2.97 (**Figure 4**). The sensitivity analysis showed the same effect size as the primary pooled effect. Due to the few included studies (n = 3), we could not generate a funnel plot to analyse publication bias. However, Egger’s regression test showed no publication bias (*P* = 0.506).

### Effect on global cognitive function

Five randomised controlled trials investigated the effectiveness of global cognitive function. The pooled Hedge's g was 0.30 (95% CI = 0.02, 0.58), and we observed low heterogeneity (Q-statistic = 2.63, *I^2^* = 0; *P* = 0.621) (**Figure 5**). We found no significant difference between the sensitivity test and main analyses (Hedges' g = 0.30; 95% CI = 0.02, 0.58). We could not analyse publication bias through a funnel plot due to the low number of included studies (n = 10). The Egger test revealed no publication bias (*P* = 0.525).

### Subgroup analysis on overall attention

We performed subgroup analyses on the intervention characteristics in cognitive training toward the primary outcome of overall attention and found that the individual-focused intervention format was superior to the group format (Hedges’ g = 0.44; 95% CI = 0.05, 0.84), the <60-minute length of training format was superior to the ≥60 minutes (Hedges’ g = 0.97; 95% CI = 0.09, 1.85), <3 weekly training sessions was better than ≥3 sessions (Hedges’ g = 0.63; 95% CI = 0.09, 1.17), a total training of ≥8 weeks was superior to <8 weeks (Hedges’ g = 0.52; 95% CI 0.12 to 0.92), and the total session lasting <24 weeks was superior to ≥24 weeks (Hedges’ g = 0.30; 95% CI = 0.08, 0.52) (Figure S2 and Table S5 in the **Online Supplementary Document**).

### Meta-regression analysis of the characteristics of overall attention

We performed a meta-regression analysis for the outcome of overall attention; the resulting variables of mean age (β = 0.049; 95% CI = -0.10, 0.08) showed non-significant relationships with our results.

## DISCUSSION

This meta-analysis is the first to assess the effect size of cognitive training on attention domains for older adults with mild cognitive impairment and mild dementia. We found that, in elderly with mild cognitive impairment and mild dementia, cognitive training exerts a small-to-medium effect overall attention, selective attention, divided attention, and global cognitive function, which is inconsistent with a previous network meta-analysis [36] on the effects of different cognitive interventions on cognitive outcomes in individuals with mild cognitive impairment. Despite some positive results from individual studies, the pooled results indicated that no intervention was effective in improving overall cognition or attention. However, our findings are consistent with another meta-analysis [37] on different interventions, which may explain the discrepancy in our findings on overall cognitive function. Additionally, because there is currently a lack of comparable meta-analyses specifically targeting attention in elderly individuals with mild cognitive impairment and because various methods can be used to assess different aspects of attention, future research should more extensively discuss the impact of attention. While the scientific study of attention originated in psychology, the different underlying mechanisms of these behavioural patterns are yet to be determined.

Our subgroup findings suggest cognitive training on attention showed better effectiveness with individual format, a length of training <60 minutes, <3 weekly training sessions, a total training of ≥8 weeks, and a total of <24 weeks of session for mild cognitive impairment and mild dementia. The effect of cognitive training on overall attention was lower in older people by 0.0097, but was not significant. The effect was reduced by 0.0097 as the participants’ age increased. As age increases, attention tends to be more easily distracted, so based on research findings, it is recommended that the training duration should not exceed 60 minutes. Conducting training sessions individually and extending the training can enhance the effectiveness of the training. This suggests that each training session should be conducted individually, with a duration of less than 60 minutes and that extending the duration of the training weeks can make the training more effective.

### Strengths and limitations

Our meta-analysis has several strengths. It is the first to synthesise the evidence regarding the impact of cognitive training on attention in elderly individuals with mild cognitive impairment. Furthermore, we conducted a comprehensive search without language restrictions to identify eligible studies while adhering to the PRISMA statement checklist, and registered the study protocol with PROSPERO to enhance the transparency and traceability of the study, ensuring its accuracy and reliability. Due to the lack of evidence regarding the impact of cognitive training on attention in elderly individuals with mild cognitive impairment, this study also contributes to a more comprehensive research evidence base and helps reduce publication bias and selective reporting.

However, our study has limitations. Some studies had different randomisation designs, sampling methods, types of interventions, intervention duration and frequency of practice, and measurement tools, which may have indirectly affected its results. However, as most studies focus on short-term cognitive outcomes, there is insufficient data to evaluate the persistence of cognitive training effects, which typically requires long-term follow-up and large sample sizes to detect subtle effects on function. Additionally, current meta-analyses primarily focus on older adults with mild cognitive impairment and mild dementia. However, physically healthier older adults, due to slower decline, are more likely to engage in various activities. Subsequent research could target healthier older individuals or different populations to improve the generalisability of research findings. Future studies could also include different countries, as geographical/contextual risks may lead to delayed diagnosis and uneven distribution of resources, thereby impacting patient care and outcomes. Therefore, improving fairness and accessibility in the diagnosis and management of mild cognitive impairment globally remains an important goal. Few studies reported the variables we sought to examine to explain the heterogeneity, so we could only investigate a subset of prospective moderator variables.

## CONCLUSIONS

Our findings suggest that interventions with attention training programmes can improve partial attention functions in elderly people with mild cognitive impairment. Attention training should also be incorporated into routine activities as part of a long-term, sustained planning to delay the degradation of attention functions in the elderly. This not only reduces their risk of abnormal events (such as falls) in daily life but also improves their quality of life and helps reduce the progression of cognitive impairment, achieving early detection for secondary prevention.

## Additional material


Online Supplementary Document

## References

[1] Maresova P Hruska J Klimova B Barakovic S Krejcar O Activities of Daily Living and Associated Costs in the Most Widespread Neurodegenerative Diseases: A Systematic Review. Clin Interv Aging. 2020;15:1841-62. 10.2147/CIA.S264688 33061334 PMC7538005

[2] Petersen RC Lopez O Armstrong MJ Getchius TSD Ganguli M Gloss D Practice guideline update summary: Mild cognitive impairment: Report of the Guideline Development, Dissemination, and Implementation Subcommittee of the American Academy of Neurology. Neurology. 2018;90:126-35. 10.1212/WNL.0000000000004826 29282327 PMC5772157

[3] Bazgir B Shamseddini A Hogg JA Ghadiri F Bahmani M Diekfuss JA Is cognitive control of perception and action via attentional focus moderated by motor imagery? BMC Psychol. 2023;11:12. 10.1186/s40359-023-01047-z 36647147 PMC9841651

[4] Chan PT Chang WC Chiu HL Kao CC Liu D Chu H Effect of interactive cognitive-motor training on eye-hand coordination and cognitive function in older adults. BMC Geriatr. 2019;19:27. 10.1186/s12877-019-1029-y 30691404 PMC6350349

[5] Chiu HL Chan PT Kao CC Chu H Chang PC Hsiao SS Effectiveness of executive function training on mental set shifting, working memory and inhibition in healthy older adults: A double-blind randomized controlled trials. J Adv Nurs. 2018;74:1099-113. 10.1111/jan.13519 29288507

[6] Yang HL Chu H Kao CC Chiu HL Tseng IJ Tseng P Development and effectiveness of virtual interactive working memory training for older people with mild cognitive impairment: a single-blind randomised controlled trial. Age Ageing. 2019;48:519-25. 10.1093/ageing/afz029 30989165

[7] Yang HL Chu H Miao NF Chang PC Tseng P Chen R The Construction and Evaluation of Executive Attention Training to Improve Selective Attention, Focused Attention, and Divided Attention for Older Adults With Mild Cognitive Impairment: A Randomized Controlled Trial. Am J Geriatr Psychiatry. 2019;27:1257-67. 10.1016/j.jagp.2019.05.017 31248769

[8] Yang HL Chu H Kao CC Miao NF Chang PC Tseng P Construction and evaluation of multidomain attention training to improve alertness attention, sustained attention, and visual-spatial attention in older adults with mild cognitive impairment: A randomized controlled trial. Int J Geriatr Psychiatry. 2020;35:537-46. 10.1002/gps.5269 31994767

[9] Belleville S Mellaha S Boller B Ouellet É Activation changes induced by cognitive training are consistent with improved cognitive reserve in older adults with subjective cognitive decline. Neurobiol Aging. 2023;121:107-18. 10.1016/j.neurobiolaging.2022.10.010 36401900

[10] Joseph HMLN Fisher N Novick DR Gibson C Rothenberger SD Foust JE Research Review: A systematic review and meta-analysis of infant and toddler temperament as predictors of childhood attention-deficit/hyperactivity disorder. J Child Psychol Psychiatry. 2023;64:715-735. 10.1111/jcpp.13753 36599815 PMC10404471

[11] Wang M Yang X Yu J Zhu J Kim HD Cruz A Effects of Physical Activity on Inhibitory Function in Children with Attention Deficit Hyperactivity Disorder: A Systematic Review and Meta-Analysis. Int J Environ Res Public Health. 2023;20:1032. 10.3390/ijerph20021032 36673793 PMC9859519

[12] Nejati V Derakhshan Z Mohtasham A The effect of comprehensive working memory training on executive functions and behavioral symptoms in children with attention deficit-hyperactivity disorder (ADHD). Asian J Psychiatr. 2023;81:103469. 10.1016/j.ajp.2023.103469 36669291

[13] Higgins JPTTJ, Chandler J, Cumpston M, Li T, Page MJ, Welch VA, editors. Cochrane Handbook for Systematic Reviews of Interventions, 2nd Edition. Chichester: John Wiley & Sons; 2019.

[14] Page MJ McKenzie JE Bossuyt PM Boutron I Hoffmann TC Mulrow CD The PRISMA 2020 statement: an updated guideline for reporting systematic reviews. BMJ. 2021;372:n71. 10.1136/bmj.n71 33782057 PMC8005924

[15] Higgins JPAD Gøtzsche PC Jüni P Moher D Oxman AD Savovic J Cochrane Bias Methods Cochrane Statistical Methods Group The Cochrane Collaboration’s tool for assessing risk of bias in randomised trials. BMJ. 2011;343:d5928. 10.1136/bmj.d5928 22008217 PMC3196245

[16] Borenstein M. Comprehensive Meta-Analysis Software. In: Egger M, Higgins JPT, Smith GD. Systematic Reviews in Health Research. Chichester: John Wiley & Sons; 2022. p. 535-48.

[17] Borenstein M, Hedges, LV, Higgins, JP, Rothstein, HR. Introduction to meta-analysis. Chichester: John Wiley & Sons; 2011.

[18] Cumming G. Understanding the new statistics: Effect sizes, confidence intervals, and meta-analysis. New York: Routledge; 2012.

[19] Lakens D Calculating and reporting effect sizes to facilitate cumulative science: a practical primer for t-tests and ANOVAs. Front Psychol. 2013;4:863. 10.3389/fpsyg.2013.00863 24324449 PMC3840331

[20] Higgins JP Thompson SG Deeks JJ Altman DG Measuring inconsistency in meta-analyses. BMJ. 2003;327:557-60. 10.1136/bmj.327.7414.557 12958120 PMC192859

[21] Higgins J Thompson SG Deeks JJ Altman DG Statistical heterogeneity in systematic reviews of clinical trials: a critical appraisal of guidelines and practice. J Health Serv Res Policy. 2002;7:51-61. 10.1258/1355819021927674 11822262

[22] Duff K Ying J Suhrie KR Dalley BCA Atkinson TJ Porter SM Computerized Cognitive Training in Amnestic Mild Cognitive Impairment: A Randomized Clinical Trial. J Geriatr Psychiatry Neurol. 2022;35:400-9. 10.1177/08919887211006472 33783254 PMC12860473

[23] Feng H Li G Xu C Ju C Qiu X Training Rehabilitation as an Effective Treatment for Patients With Vascular Cognitive Impairment With No Dementia. Rehabil Nurs. 2017;42:290-7. 10.1002/rnj.271 27118716

[24] Fiatarone Singh MA Gates N Saigal N Wilson GC Meiklejohn J Brodaty H The Study of Mental and Resistance Training (SMART) study-resistance training and/or cognitive training in mild cognitive impairment: a randomized, double-blind, double-sham controlled trial. J Am Med Dir Assoc. 2014;15:873-80. 10.1016/j.jamda.2014.09.010 25444575

[25] Finn M McDonald S Computerised Cognitive Training for Older Persons With Mild Cognitive Impairment: A Pilot Study Using a Randomised Controlled Trial Design. Brain Impair. 2011;12:187-99. 10.1375/brim.12.3.187

[26] Finn M McDonald S Repetition-lag training to improve recollection memory in older people with amnestic mild cognitive impairment. A randomized controlled trial. Neuropsychol Dev Cogn B Aging Neuropsychol Cogn. 2015;22:244-58. 10.1080/13825585.2014.915918 24820545

[27] Gagnon LG Belleville S Training of attentional control in mild cognitive impairment with executive deficits: results from a double-blind randomised controlled study. Neuropsychol Rehabil. 2012;22:809-35. 10.1080/09602011.2012.691044 22712452

[28] Li BY He NY Qiao Y Xu HM Lu YZ Cui PJ Computerized cognitive training for Chinese mild cognitive impairment patients: A neuropsychological and fMRI study. Neuroimage Clin. 2019;22:101691. 10.1016/j.nicl.2019.101691 30708349 PMC6354286

[29] Pantoni L Poggesi A Diciotti S Valenti R Orsolini S Della Rocca E Effect of Attention Training in Mild Cognitive Impairment Patients with Subcortical Vascular Changes: The RehAtt Study. J Alzheimers Dis. 2017;60:615-24. 10.3233/JAD-170428 28869475 PMC5611829

[30] Torpil B Sahin S Pekcetin S Uyanik M The Effectiveness of a Virtual Reality-Based Intervention on Cognitive Functions in Older Adults with Mild Cognitive Impairment: A Single-Blind Randomized Controlled Trial Games Health J. 2021;10:109-14. 10.1089/g4h.2020.0086 33058735

[31] Vidovich MR Lautenschlager NT Flicker L Clare L McCaul K Almeida OP The PACE study: a randomized clinical trial of cognitive activity strategy training for older people with mild cognitive impairment. Am J Geriatr Psychiatry. 2015;23:360-72. 10.1016/j.jagp.2014.04.002 24801607

[32] Yu J Rawtaer I Feng L Fam J Kumar AP Kee-Mun Cheah I Mindfulness intervention for mild cognitive impairment led to attention-related improvements and neuroplastic changes: Results from a 9-month randomized control trial. J Psychiatr Res. 2021;135:203-11. 10.1016/j.jpsychires.2021.01.032 33497874

[33] Barnes DE Yaffe K Belfor N Jagust WJ DeCarli C Reed BR Computer-based cognitive training for mild cognitive impairment: results from a pilot randomized, controlled trial. Alzheimer Dis Assoc Disord. 2009;23:205-10. 10.1097/WAD.0b013e31819c6137 19812460 PMC2760033

[34] Bruderer-Hofstetter M Meichtry A Münzer T Niedermann K Effective multicomponent interventions in comparison to active control and no interventions on physical capacity, cognitive function and instrumental activities of daily living in elderly people with and without mild impaired cognition – A Systematic Review and Network meta-analysis. Ageing Res Rev. 2018;45:1-14. 10.1016/j.arr.2018.04.002 29679658

[35] Salzman T Sarquis-Adamson Y Son S Montero-Odasso M Fraser S Associations of Multidomain Interventions With Improvements in Cognition in Mild Cognitive Impairment: A Systematic Review and Meta-analysis. JAMA Netw Open. 2022;5:e226744. 10.1001/jamanetworkopen.2022.6744 35503222 PMC9066287

[36] Bernini S Panzarasa S Barbieri M Sinforiani E Quaglini S Tassorelli C A double-blind randomized controlled trial of the efficacy of cognitive training delivered using two different methods in mild cognitive impairment in Parkinson’s disease: preliminary report of benefits associated with the use of a computerized tool. Aging Clin Exp Res. 2021;33:1567-75. 10.1007/s40520-020-01665-2 32895890

[37] Biasutti M Mangiacotti A Assessing a cognitive music training for older participants: a randomised controlled trial. Int J Geriatr Psychiatry. 2018;33:271-8. 10.1002/gps.4721 28401595

[38] Doshi K Henderson SL Fan Q Wong KF Lim J Mindfulness-Based Training Does Not Improve Neuropsychological Outcomes in Mild Cognitive Impairment More Than Spontaneous Reversion Rates: A Randomized Controlled Trial. J Alzheimers Dis. 2021;84:449-58. 10.3233/JAD-215035 34542079

