# Supplementary material for: The effect of cognitive training on domains of attention in older adults with mild cognitive impairment and mild dementia: A meta-analysis of randomised controlled trials
Source: J Glob Health. 2023 Jun 30;13:04078. doi: 10.7189/jogh.13.04078 (PMC10312045; doi:10.7189/jogh.13.04078)
Supplement: Online Supplementary Document [file jogh-13-04078-s001.pdf]

Supporting information

Table S1. Electronic Database Searching Strategy

Electronic Searching Strategy		
PubMed search 71		
Population	#1	((((((((((("mild cognitive impairment"[Title/Abstract]) OR ("MCI"[Title/Abstract])) OR ("cognitive impairment"[Title/Abstract])) OR ("cognitive dysfunction"[Title/Abstract])) OR ("cognitive decline"[Title/Abstract])) OR ("cognitive disorders*"[Title/Abstract])) OR ("early stage dementia"[Title/Abstract])) OR (" Mild dementia"[Title/Abstract])) OR ("memory disorder"[Title/Abstract])) OR ("memory impairment"[Title/Abstract]) AND ((randomized controlled trial[Filter]) AND (humans[Filter]) AND (aged[Filter])))
Intervention	#2	((((((((((((((((((((((("cognitive training"[Title/Abstract]) OR ("cognitive based training"[Title/Abstract])) OR ("cognitive intervention"[Title/Abstract])) OR ("cognition-focused interventions"[Title/Abstract])) OR ("cognitive rehabilitation"[Title/Abstract])) OR ("cognitive stimulation"[Title/Abstract])) OR ("computerized cognitive training"[Title/Abstract])) OR ("computer-based cognitive training"[Title/Abstract])) OR ("computerized intervention"[Title/Abstract])) OR ("working memory training"[Title/Abstract])) OR ("memory training"[Title/Abstract])) OR ("working memory intervention"[Title/Abstract])) OR ("memory intervention"[Title/Abstract])) OR ("memory rehabilitation"[Title/Abstract])) OR ("attention training"[Title/Abstract])) OR ("attention intervention"[Title/Abstract])) OR ("attention rehabilitation"[Title/Abstract])) OR ("processing speed training"[Title/Abstract])) OR ("speed of processing training"[Title/Abstract])) OR ("speed of processing intervention"[Title/Abstract])) OR ("executive function training"[Title/Abstract])) OR ("executive function intervention"[Title/Abstract])) OR ("executive function rehabilitation"[Title/Abstract]) AND ((randomized controlled trial[Filter]) AND (humans[Filter]) AND (aged[Filter]))))
Outcomes	#3	(((((((("Attention"[Title/Abstract])) OR ("Alertness attention"[Title/Abstract])) OR ("Sustained attention"[Title/Abstract])) OR ("Selective attention"[Title/Abstract])) OR ("Focused attention"[Title/Abstract])) OR ("Divided attention"[Title/Abstract])) OR ("visual-spatial attention"[Title/Abstract]) AND ((randomized controlled trial[Filter]) AND (humans[Filter]) AND (aged[Filter])))
Search algorithm	#4	#1 AND #2 AND #3
Embase search 54		

Population	#1	(('mild cognitive impairment'/exp OR 'mild cognitive impairment' OR 'mci':ti,ab,kw OR 'cognitive impairment':ti,ab,kw OR 'cognitive dysfunction':ti,ab,kw OR 'cognitive decline':ti,ab,kw OR 'cognitive disorders*':ti,ab,kw OR 'early stage dementia':ti,ab,kw OR 'mild dementia':ti,ab,kw OR 'memory disorder':ti,ab,kw OR 'memory impairment':ti,ab,kw) AND [randomized controlled trial]/lim)
Intervention	#2	(('cognitive training'/exp OR 'cognitive training' OR 'cognitive based training':ti,ab,kw OR 'cognitive intervention':ti,ab,kw OR 'cognition-focused interventions':ti,ab,kw OR 'cognitive rehabilitation':ti,ab,kw OR 'cognitive stimulation':ti,ab,kw OR 'computerized cognitive training':ti,ab,kw OR 'computer-based cognitive training':ti,ab,kw OR 'computerized intervention':ti,ab,kw OR 'working memory training':ti,ab,kw OR 'memory training':ti,ab,kw OR 'working memory intervention':ti,ab,kw OR 'memory intervention':ti,ab,kw OR 'memory rehabilitation':ti,ab,kw OR 'attention training':ti,ab,kw OR 'attention intervention':ti,ab,kw OR 'attention rehabilitation':ti,ab,kw OR 'processing speed training':ti,ab,kw OR 'speed of processing training':ti,ab,kw OR 'speed of processing intervention':ti,ab,kw OR 'executive function training':ti,ab,kw OR 'executive function intervention':ti,ab,kw OR 'executive function rehabilitation':ti,ab,kw) AND [randomized controlled trial]/lim)
Outcomes	#3	(('attention'/exp OR 'attention' OR 'alertness attention':ti,ab,kw OR 'sustained attention':ti,ab,kw OR 'selective attention':ti,ab,kw OR 'focused attention':ti,ab,kw OR 'divided attention':ti,ab,kw OR 'visual-spatial attention':ti,ab,kw) AND [randomized controlled trial]/lim)
Filters	#4	('older adults'/exp OR 'older adults') AND [randomized controlled trial]/lim)
Search algorithm	#5	#1 AND #2 AND #3 AND #4
Web of Science 11		
Population	#1	"mild cognitive impairment" (Abstract) OR "MCI" (Abstract) OR "cognitive impairment" (Abstract) OR "cognitive dysfunction" (Abstract) OR "cognitive decline" (Abstract) OR "cognitive disorders*" (Abstract) OR "early stage dementia" (Abstract) OR " Mild dementia" (Abstract) OR "memory disorder" (Abstract) OR "memory impairment" (Abstract)
Intervention	#2	"cognitive training" (Abstract) OR "cognitive based training" (Abstract) OR "cognitive intervention" (Abstract) OR "cognition-focused interventions" (Abstract) OR "cognitive rehabilitation" (Abstract) OR "cognitive stimulation" (Abstract) OR "computerized cognitive training" (Abstract) OR "computer-based cognitive training" (Abstract) OR "computerized intervention" (Abstract) OR "working memory training" (Abstract) OR "memory training" (Abstract) OR "working memory intervention" (Abstract) OR "memory intervention" (Abstract) OR "memory rehabilitation" (Abstract) OR "attention training" (Abstract) OR "attention intervention" (Abstract) OR "attention rehabilitation" (Abstract) OR"processing speed training"(Abstract) OR "speed of processing training"(Abstract) OR "speed of processing intervention"(Abstract) OR "executive function training"(Abstract) OR "executive function intervention"(Abstract) OR "executive function

		rehabilitation”(Abstract) AND“Attention” (Abstract) OR “Alertness attention”(Abstract) OR “Sustained attention”(Abstract) OR “Selective attention”(Abstract) OR “Focused attention” (Abstract) OR “Divided attention”(Abstract) OR “visual-spatial attention”(Abstract) AND "randomized controlled trial" (Abstract) AND Older Adults (Abstract)
Outcomes	#3	“Attention” (Abstract) OR “Alertness attention”(Abstract) OR “Sustained attention”(Abstract) OR “Selective attention”(Abstract) OR “Focused attention” (Abstract) OR “Divided attention”(Abstract) OR “visual-spatial attention”(Abstract) AND "randomized controlled trial" (Abstract) AND Older Adults (Abstract)
Filters	#4	"randomized controlled trial" (Abstract) AND Older Adults (Abstract)
Search algorithm	#5	#1 AND #2 AND #3 AND #4
Scopus 86		
Population	#1	(((((((((("mild cognitive impairment"[Title/Abstract]) OR ("MCI"[Title/Abstract])) OR ("cognitive impairment"[Title/Abstract])) OR ("cognitive dysfunction"[Title/Abstract])) OR ("cognitive decline"[Title/Abstract])) OR ("cognitive disorders*"[Title/Abstract])) OR ("early stage dementia"[Title/Abstract])) OR (" Mild dementia"[Title/Abstract])) OR ("memory disorder"[Title/Abstract])) OR ("memory impairment"[Title/Abstract]) AND ((randomized controlled trial[Filter]) AND (humans[Filter]) AND (aged[Filter])))
Intervention	#2	((((((((((((((((((((("cognitive training"[Title/Abstract]) OR ("cognitive based training"[Title/Abstract])) OR ("cognitive intervention"[Title/Abstract])) OR ("cognition-focused interventions"[Title/Abstract])) OR ("cognitive rehabilitation"[Title/Abstract])) OR ("cognitive stimulation"[Title/Abstract])) OR ("computerized cognitive training"[Title/Abstract])) OR ("computer-based cognitive training"[Title/Abstract])) OR ("computerized intervention"[Title/Abstract])) OR ("working memory training"[Title/Abstract])) OR ("memory training"[Title/Abstract])) OR ("working memory intervention"[Title/Abstract])) OR ("memory intervention"[Title/Abstract])) OR ("memory rehabilitation"[Title/Abstract])) OR ("attention training"[Title/Abstract])) OR ("attention intervention"[Title/Abstract])) OR ("attention rehabilitation"[Title/Abstract])) OR ("processing speed training"[Title/Abstract])) OR ("speed of processing training"[Title/Abstract])) OR ("speed of processing intervention"[Title/Abstract])) OR ("executive function training"[Title/Abstract])) OR ("executive function intervention"[Title/Abstract])) OR ("executive function rehabilitation"[Title/Abstract]) AND ((randomized controlled trial[Filter]) AND (humans[Filter]) AND (aged[Filter]))))
	#3	((((((("Attention"[Title/Abstract])) OR ("Alertness attention"[Title/Abstract])) OR ("Sustained attention"[Title/Abstract])) OR ("Selective

		attention"[Title/Abstract])) OR ("Focused attention"[Title/Abstract])) OR ("Divided attention"[Title/Abstract])) OR ("visual-spatial attention"[Title/Abstract]) AND ((randomized controlled trial[Filter]) AND (humans[Filter]) AND (aged[Filter])))
Search algorithm	#4	#1 AND #2 AND #3
CINAHL 1		
Population	#1	((((((((((("mild cognitive impairment"[Title/Abstract]) OR ("MCI"[Title/Abstract])) OR ("cognitive impairment"[Title/Abstract])) OR ("cognitive dysfunction"[Title/Abstract])) OR ("cognitive decline"[Title/Abstract])) OR ("cognitive disorders*"[Title/Abstract])) OR ("early stage dementia"[Title/Abstract])) OR (" Mild dementia"[Title/Abstract])) OR ("memory disorder"[Title/Abstract])) OR ("memory impairment"[Title/Abstract]) AND ((randomized controlled trial[Filter]) AND (humans[Filter]) AND (aged[Filter])))
Intervention	#2	((((((((((((((((((((((("cognitive training"[Title/Abstract]) OR ("cognitive based training"[Title/Abstract])) OR ("cognitive intervention"[Title/Abstract])) OR ("cognition-focused interventions"[Title/Abstract])) OR ("cognitive rehabilitation"[Title/Abstract])) OR ("cognitive stimulation"[Title/Abstract])) OR ("computerized cognitive training"[Title/Abstract])) OR ("computer-based cognitive training"[Title/Abstract])) OR ("computerized intervention"[Title/Abstract])) OR ("working memory training"[Title/Abstract])) OR ("memory training"[Title/Abstract])) OR ("working memory intervention"[Title/Abstract])) OR ("memory intervention"[Title/Abstract])) OR ("memory rehabilitation"[Title/Abstract])) OR ("attention training"[Title/Abstract])) OR ("attention intervention"[Title/Abstract])) OR ("attention rehabilitation"[Title/Abstract])) OR ("processing speed training"[Title/Abstract])) OR ("speed of processing training"[Title/Abstract])) OR ("speed of processing intervention"[Title/Abstract])) OR ("executive function training"[Title/Abstract])) OR ("executive function intervention"[Title/Abstract])) OR ("executive function rehabilitation"[Title/Abstract]) AND ((randomized controlled trial[Filter]) AND (humans[Filter]) AND (aged[Filter]))))
Filters	#3	(((((((("Attention"[Title/Abstract])) OR ("Alertness attention"[Title/Abstract])) OR ("Sustained attention"[Title/Abstract])) OR ("Selective attention"[Title/Abstract])) OR ("Focused attention"[Title/Abstract])) OR ("Divided attention"[Title/Abstract])) OR ("visual-spatial attention"[Title/Abstract]) AND ((randomized controlled trial[Filter]) AND (humans[Filter]) AND (aged[Filter])))

Search algorithm	#4	#1 AND #2 AND #3
PsycINFO 5		
Population	#1	(((((((((("mild cognitive impairment"[Title/Abstract]) OR ("MCI"[Title/Abstract])) OR ("cognitive impairment"[Title/Abstract])) OR ("cognitive dysfunction"[Title/Abstract])) OR ("cognitive decline"[Title/Abstract])) OR ("cognitive disorders*"[Title/Abstract])) OR ("early stage dementia"[Title/Abstract])) OR (" Mild dementia"[Title/Abstract])) OR ("memory disorder"[Title/Abstract])) OR ("memory impairment"[Title/Abstract]) AND ((randomized controlled trial[Filter]) AND (humans[Filter]) AND (aged[Filter])))
Intervention	#2	((((((((((((((((((((("cognitive training"[Title/Abstract]) OR ("cognitive based training"[Title/Abstract])) OR ("cognitive intervention"[Title/Abstract])) OR ("cognition-focused interventions"[Title/Abstract])) OR ("cognitive rehabilitation"[Title/Abstract])) OR ("cognitive stimulation"[Title/Abstract])) OR ("computerized cognitive training"[Title/Abstract])) OR ("computer-based cognitive training"[Title/Abstract])) OR ("computerized intervention"[Title/Abstract])) OR ("working memory training"[Title/Abstract])) OR ("memory training"[Title/Abstract])) OR ("working memory intervention"[Title/Abstract])) OR ("memory intervention"[Title/Abstract])) OR ("memory rehabilitation"[Title/Abstract])) OR ("attention training"[Title/Abstract])) OR ("attention intervention"[Title/Abstract])) OR ("attention rehabilitation"[Title/Abstract])) OR ("processing speed training"[Title/Abstract])) OR ("speed of processing training"[Title/Abstract])) OR ("speed of processing intervention"[Title/Abstract])) OR ("executive function training"[Title/Abstract])) OR ("executive function intervention"[Title/Abstract])) OR ("executive function rehabilitation"[Title/Abstract]) AND ((randomized controlled trial[Filter]) AND (humans[Filter]) AND (aged[Filter]))))
Filters	#3	((((((("Attention"[Title/Abstract])) OR ("Alertness attention"[Title/Abstract])) OR ("Sustained attention"[Title/Abstract])) OR ("Selective attention"[Title/Abstract])) OR ("Focused attention"[Title/Abstract])) OR ("Divided attention"[Title/Abstract])) OR ("visual-spatial attention"[Title/Abstract]) AND ((randomized controlled trial [Filter]) AND (humans[Filter]) AND (aged[Filter])))
Search algorithm	#4	#1 AND #2 AND #3
Cochrane Library 58		
Population	#1	Mild Cognitive Impairment
Intervention	#2	Cognitive Training
Outcomes	#3	Attention

Filters	#4	Randomized controlled trial

Table S2. List of the included studies

1	Bernini S, Panzarasa S, Barbieri M, Sinforiani E, Quaglini S, Tassorelli C, et al. A double-blind randomized controlled trial of the efficacy of cognitive training delivered using two different methods in mild cognitive impairment in Parkinson's disease: preliminary report of benefits associated with the use of a computerized tool. Aging Clin Exp Res. 2021;33:1567-75.
2	Biasutti M, Mangiacotti A. Assessing a cognitive music training for older participants: a randomised controlled trial. Int J Geriatr Psychiatry. 2018;33:271-8.
3	Doshi K, Henderson SL, Fan Q, Wong KF, Lim J. Mindfulness-Based Training Does Not Improve Neuropsychological Outcomes in Mild Cognitive Impairment More Than Spontaneous Reversion Rates: A Randomized Controlled Trial. J Alzheimers Dis. 2021;84:449-58.
4	Duff K, Ying J, Suhrie KR, Dalley BCA, Atkinson TJ, Porter SM, et al. Computerized Cognitive Training in Amnestic Mild Cognitive Impairment: A Randomized Clinical Trial. J Geriatr Psychiatry Neurol. 2022;35:400-9.
5	Feng H, Li G, Xu C, Ju C, Qiu X. Training Rehabilitation as an Effective Treatment for Patients With Vascular Cognitive Impairment With No Dementia. Rehabil Nurs. 2017;42:290-7.
6	Fiatarone Singh MA, Gates N, Saigal N, Wilson GC, Meiklejohn J, Brodaty H, et al. The Study of Mental and Resistance Training (SMART) study-resistance training and/or cognitive training in mild cognitive impairment: a randomized, double-blind, double-sham controlled trial. J Am Med Dir Assoc. 2014;15:873-80.
7	Finn M, McDonald S. Computerised Cognitive Training for Older Persons With Mild Cognitive Impairment: A Pilot Study Using a Randomised Controlled Trial Design. Brain Impair. 2011;12(3):187–99.
8	Finn M, McDonald S. Repetition-lag training to improve recollection memory in older people with amnestic mild cognitive impairment. A randomized controlled trial. Neuropsychol Dev Cogn B Aging Neuropsychol Cogn. 2015;22:244-58.
9	Gagnon LG, Belleville S. Training of attentional control in mild cognitive impairment with executive deficits: results from a double-blind randomised controlled study. Neuropsychol Rehabil. 2012;22:809-35.
10	Li BY, He NY, Qiao Y, Xu HM, Lu YZ, Cui PJ, et al. Computerized cognitive training for Chinese mild cognitive impairment patients: A neuropsychological and fMRI study. Neuroimage Clin. 2019;22:101691.
11	Pantoni L, Poggesi A, Diciotti S, Valenti R, Orsolini S, Della Rocca E, et al. Effect of Attention Training in Mild Cognitive Impairment Patients with Subcortical Vascular Changes: The RehAtt Study. J Alzheimers Dis. 2017;60:615-24.
12	Torpil B, Sahin S, Pekcetin S, Uyanik M. The Effectiveness of a Virtual Reality-Based Intervention on Cognitive Functions in Older Adults with Mild Cognitive Impairment: A Single-Blind, Randomized Controlled Trial. Games Health J. 2021;10:109-14.
13	Vidovich MR, Lautenschlager NT, Flicker L, Clare L, McCaul K, Almeida OP. The PACE study: a randomized clinical trial of cognitive activity strategy training for older people with mild cognitive impairment. Am J Geriatr Psychiatry. 2015;23:360-72.
14	Yang HL, Chu H, Kao CC, Miao NF, Chang PC, Tseng P, et al. Construction and evaluation of multidomain attention training to improve alertness attention, sustained attention, and visual-spatial attention in older adults with mild cognitive impairment: A randomized controlled trial. Int J Geriatr Psychiatry. 2020;35:537-46.

15 Yang HL, Chu H, Miao NF, Chang PC, Tseng P, Chen R, et al. The Construction and Evaluation of Executive Attention Training to Improve Selective Attention, Focused Attention, and Divided Attention for Older Adults With Mild Cognitive Impairment: A Randomized Controlled Trial. Am J Geriatr Psychiatry. 2019;27:1257-67.

16 Yu J, Rawtaer I, Feng L, Fam J, Kumar AP, Kee-Mun Cheah I, et al. Mindfulness intervention for mild cognitive impairment led to attention-related improvements and neuroplastic changes: Results from a 9-month randomized control trial. J Psychiatr Res. 2021;135:203-11.

17 Barnes DE, Yaffe K, Belfor N, Jagust WJ, DeCarli C, Reed BR, et al. Computer-based cognitive training for mild cognitive impairment: results from a pilot randomized, controlled trial. Alzheimer Dis Assoc Disord. 2009;23:205-10.

Table S3. Risk of bias assessment

Study	Randomization process	Deviations from intended interventions	Missing outcome data	Measurement of the outcome	Selection of the reported result	Overall Bias
Bernini et al., 2020	S	L	L	L	L	S
Biasutti et al., 2017	S	L	L	L	L	S
Doshi et al., 2021	S	L	L	L	L	S
Duff et al., 2022	L	L	L	L	L	L
Feng et al., 2016	S	S	L	L	L	S
Fiatarone Singh et al., 2014	L	L	L	L	L	L
Finn et al., 2011	L	L	L	L	L	L
Finn et al., 2015	L	L	L	L	L	L
Gagnon et al., 2012	S	L	L	L	L	S
Li et al., 2019	S	L	L	L	L	S
Pantoni et al., 2017	S	L	L	L	L	S
Torpil et al., 2021	S	L	L	L	L	S
Vidovich et al., 2015	S	L	L	L	L	S
Yang et al., 2020	L	L	L	L	L	L
Yang et al., 2019	L	L	L	L	L	L
Yu et al., 2021	S	L	L	L	L	S
Barnes et al., 2009	L	L	L	L	L	L

Risk of bias judgement: Low Risk / Some Concern / High Risk

Table S4. Study characteristics

Study citation	Participants	Intervention and Intensity	Outcome indicators measurement tool	Follow-up time	Risk of Bias 2.0
Bernini et al. (2020). Italy [24]	·Total N: 48<br>·Complete N: CCT/PCT/CG: 18/12/18<br>·Mean age: 74.6(±5.7) /69.8(±9.7)/69.3(±7.7)<br>·Gender (F): 33/40/33<br>·MoCA: 19.09±2.84/ 20.83 ±3.15/19.17±3.49	·CCT: computer-based cognitive training with CoRe sofe ware<br>·PCT: paper-and-pencil cognitive training<br>·CG: unstructured Activity intervention<br>·Format: individual<br>·Frequency<br>·min. a day: 45<br>·day of week: 4<br>·total weeks: 3<br>·total sessions:12	·global cognition: MoCA<br>·attention: TMT A and B	Pretest Posttest	Some Concern
Biasutti et al. (2017). Italy [25]	·Total N: 35<br>·Complete N: EG/CG: 18/17<br>·Mean age: 83.4(±7.8)/ 83.8(±6.2)<br>·Gender (F/M): 23/12<br>·MMSE: 22.39±3.65/ 24.53±3.50	·EG: cognitive music training<br>·CG: gymnastic activities<br>·Format: group<br>·Frequency<br>·min. a day: 70<br>·day of week: bi-weekly<br>·total sessions:12	·global cognition: MMSE<br>·Selective attention: TMT-A<br>·visual selective attention: AMT	Pretest Posttest	Some Concern
Doshi et al. (2021). Singapore [26]	·Total N: 76<br>·Complete N: MBT/CRT/TAU: 32/27/17<br>·Mean age: 67.6(±5.3)/ 67.1(±3.4)/66.3(±6.7)<br>·Gender (M): 16/10/8	·MBT: mindfulness based training<br>·CRT: cognitive rehabilitation therapy<br>·TAU: treatment as usual<br>·Format: group<br>·Frequency<br>·min. a day: 2hrs<br>·day of week: 1<br>·total weeks: 8<br>·total sessions: 8	·global cognition: MoCA<br>·Attention、immediate memory、delayed memory: RBANS	Pretest Posttest	Some Concern
Duff et al. (2022). USA [27]	·Total N: 113<br>·Complete N: EG/CG: 55/58<br>·Mean age: 74.9(±6.3)/ 74.9(±5.8)<br>·Gender (F/M): 51/62	·EG: Computerized Cognitive Training<br>·CG: active Control group<br>·Format: individual<br>·Frequency<br>·min. a day: 45<br>·day of week: 4-5<br>·total weeks: 12-13<br>·total hours: 40	·Auditory Memory/ Attention Index	Pretest Posttest One year	Low Risk
Feng et al. (2016). China [28]	·Total N: 73<br>·Complete N: EG/CG: 36/37<br>·Mean age: 66.1(±13.3)/ 66.0(±14.2)<br>·Gender (F/M): 31/42	·EG: systemic cognitive training<br>·CG: routine care<br>·Format: individual<br>·Frequency<br>·min. a day: 60<br>·total weeks: 12<br>·total hours: 60	·SCWT<br>·visual attention: TMT	Pretest Posttest	Some Concern

Fiatarone Singh et al. (2014). Australian [29]	·Total N: 100<br>·Complete N: EG1/FG2/EG3/CG: 24/22/27/27<br>·Mean age: 66.1(±13.3)/ 66.0(±14.2)<br>·Gender (F/M): 31/42<br>·MMSE: 28±2/27±1/ 27±2/27±2	·EG1: CT Intervention (+Sham Exercise)<br>·EG2: Progressive resistance training: PRT (+Sham Cognitive)<br>·EG3: Combined CT and PRT<br>·CG: sham cognitive and sham exercise<br>·Format: individual<br>·Frequency<br>·min. a day:60-100<br>·day of week: 2-3<br>·total sessions: 6M	·global cognition: ADAS-Cog、CDR<br>·Attention/speed: SDMT	Pretest<br>6 and 18 months	Low Risk
Finn et al. (2011). Australian [30]	·Total N: 16<br>·Complete N: treatment/waitlist: 8/8<br>·Mean age: 69.0(±7.7)/ 76.4(±6.5)<br>·Gender (F/M): 8/8	·EG: Computerised Cognitive Training<br>·CG: control group<br>·Format: individual<br>·Frequency<br>·min. a day: 60-100<br>·total sessions: 30	·cognitive function: CANTAB<br>·Visual sustained attention: RVP	Pretest posttest	Low Risk
Finn et al. (2015). Australian [31]	·Total N: 24<br>·Complete N: EG/CG: 12/12<br>·Mean age: 72.8(±5.7)/ 75.1(±7.5)<br>·Gender (F/M): 7/17	·EG: repetition-lag training<br>·CG: no contact<br>·Format: individual<br>·Frequency<br>·day of week: 2<br>·total weeks: 4	·Attention: Number sequencing<br>·Cognitive flexibility: Number-Letter switching	Pretest Posttest	Low Risk
Gagnon et al. (2012). Canada [32]	·Total N: 24<br>·Complete N: EG/CG: 12/12<br>·Mean age: 67.0(±7.8)/ 68.4(±6.0)<br>·Gender (F/M): NA<br>·MMSE: 28.08±1.16/ 27.83±1.47<br>·MoCA: 24.83±2.04/ 23.71±1.39	·EG: computer-based training programme involving Variable Priority (VP)<br>·CG: active control group performed Fixed Priority (FP) training<br>·Format: individual<br>·Frequency<br>·min. a day: 60<br>·day of week: 3<br>·total weeks: 2<br>·total sessions: 6	·Attention: TEA、TMT A&B<br>·Divided Attention Questionnaire	Pretest Posttest	Some Concern
Li et al. (2019). China [33]	·Total N: 141<br>·Complete N: EG/CG: 78/63<br>·Mean age: 69.5(±7.3)/ 71.5(±6.8)<br>·Gender(F/M): 75/66<br>·CDR: 1.3±0.9/19.2±3.9	·EG: attention training<br>·CG: standard care<br>·Format: individual<br>·Frequency<br>·day of week: 3-4<br>·total weeks: 6M<br>·total min: 120-160	·attention and memory: ACER	Pretest<br>6 and 18 months	Some Concern
Pantoni et al (2017). Italy [34]	·Total N: 43<br>·Complete N: EG/CG: 21/22<br>·Mean age: 74.2(±6.0)/ 75.9(±7.6)	·EG: attention training<br>·CG: standard care<br>·Format: individual<br>·Frequency<br>·min. a day: 2h	·Cognitive outcomes: MoCA、RAVL、ROCF、Visual search、Symbol Digit Modalities Test、	Pretest Posttest<br>6 and 12 months	Some Concern

	·Gender (F/M): 15/28<br>·MoCA: 19.9±4.8/18.6±4.4	·day of week: 1<br>·total weeks: 20<br>·total sessions: 40h	Stroop Test、TMT A&B、Phonemic verbal fluency		
Torpil et al. (2021). Turkey [35]	·Total N: 61<br>·Complete N: EG/CG: 30/31<br>·Mean age: 70.1(±2.6)/ 70.3(±2.7)<br>·Gender (F/M): 36/25	·EG: VR+CR<br>·CG: CR<br>·Format: individual<br>·Frequency<br>·min. a day: 45<br>·day of week: 2<br>·total weeks: 12<br>·total sessions: 24	·attention/ concentration	Pretest Posttest	Some Concern
Vidovich et al. (2015). Australian [36]	·Total N: 160<br>·Complete N: EG/CG: 80/80<br>·Mean age: 75.1(±6.1)/ 74.9(±5.5)<br>·Gender (F): 51/35	·EG: cognitive activity (CA) strategy-training<br>·CG: control education group<br>·Format: group<br>·Frequency<br>·min. a day: 90<br>·day of week: 2<br>·total weeks: 5<br>·total sessions:10	·Cognitive functions: CAMCOG-R<br>·Attention: DS<br>·Attention/Proc. Speed: TMT-A	Pretest 10 weeks、52 weeks、104 weeks	Some Concern
Yang et al. (2020). Taiwan [8]	·Total N: 78<br>·Complete N: EG/CG: 39/39<br>·Mean age: 77.2(±8.1)/ 81.8(±7.1)<br>·Gender (F/M): 62/16<br>·MoCA: 22.7±3.9/21.9 ±5.7	·EG: multidomain attention training with CogniPlus (MAT)<br>·CG: active Control group (AC)<br>·Format: individual<br>·Frequency<br>·min. a day: 45<br>·day of week: 3<br>·total weeks: 6<br>·total sessions:18	·Alertness attention: TMT-B<br>·Visual-spatial attention: TMT-A<br>·Sustained attention: DVT<br>·Cognitive functions: MoCA	Pretest Posttest 3 and 6 months	Low Risk
Yang et al. (2019). Taiwan [7]	·Total N: 70<br>·Complete N: EG/CG: 35/35<br>·Mean age: 75.6(±6.9)/ 80.8(±6.7)<br>·Gender (F/M): 55/15<br>·MoCA: 23.1±3.9/21.6 ±5.8	·EG: executive attention training (EAT)<br>·CG: active Control group (AC)<br>·Format: individual<br>·Frequency<br>·min. a day: 45<br>·day of week: 3<br>·total weeks: 6<br>·total sessions:18	·Selective attention: Digit Span Task<br>·Focused attention: SCWT<br>·Divided attention: TMT-B	Pretest Posttest 3 and 6 months	Low Risk
Yu et al. (2021). Singapore [37]	·Total N: 54<br>·Complete N: EG/CG: 27/27<br>·Mean age: 71.3(±5.6)/ 71.4(±6.0)<br>·Gender (F/M): 40/14	·EG: mindfulness awareness program (MAP)<br>·CG: health education program (HEP)<br>·Format: group<br>·Frequency<br>·total weeks: 9M	·Divided attention and sets witching: Color trails test	Pretest 3 and 9 months	Some Concern
Barnes et al. (2009). USA [38]	·Total N: 47<br>·Complete N: EG/CG: 22/25<br>·Mean age: 74.1(±8.7)/ 74.8(±7.2)<br>·Gender (M%):	·EG: computer-based, cognitive training program<br>·CG: passive computer activities<br>·Format: individual	·Attention/Executive : RBANS attention、Design fluency、TMT、Spatial span	Pretest Posttest	Low Risk

59.1%/60%	·Frequency<br>·min. a day: 100<br>·day of week: 5<br>·total weeks: 6<br>·total sessions: 30	

MoCA: Montreal Overall Cognitive Assessment, TMT: Trail Making Test, MMSE: Mini-Mental State Examination, AMT: Attentional matrices test, SCWT: Stroop Color-Word test, CT: cognitive training, ADAS-Cog: Alzheimer’s Disease Assessment Scale-cognitive subscale, CDR: Clinical Dementia Rating, SDMT: Symbol Digit Modalities Test (SDMT), CANTAB: Cambridge Automated Neuropsychological Test Battery, RVP: Rapid visual information Processing, TEA: Test of Everyday Attention, ACER: Addenbrooke's Cognitive examination-revised Scores, RAVL: Rey Auditory-Verbal Learning Test, ROCF: Rey-Osterrieth Complex Figure Test, VR: Virtual reality, CR: Cognitive rehabilitation, CAMCOG-R: Cambridge Cognitive Examination-Revised, DS: Digit Span, DVT: Digit Vigilance Test and RBANS: Repeatable Battery for Assessment of Cognitive Status

Figure S1. Funnel Plot on overall attention

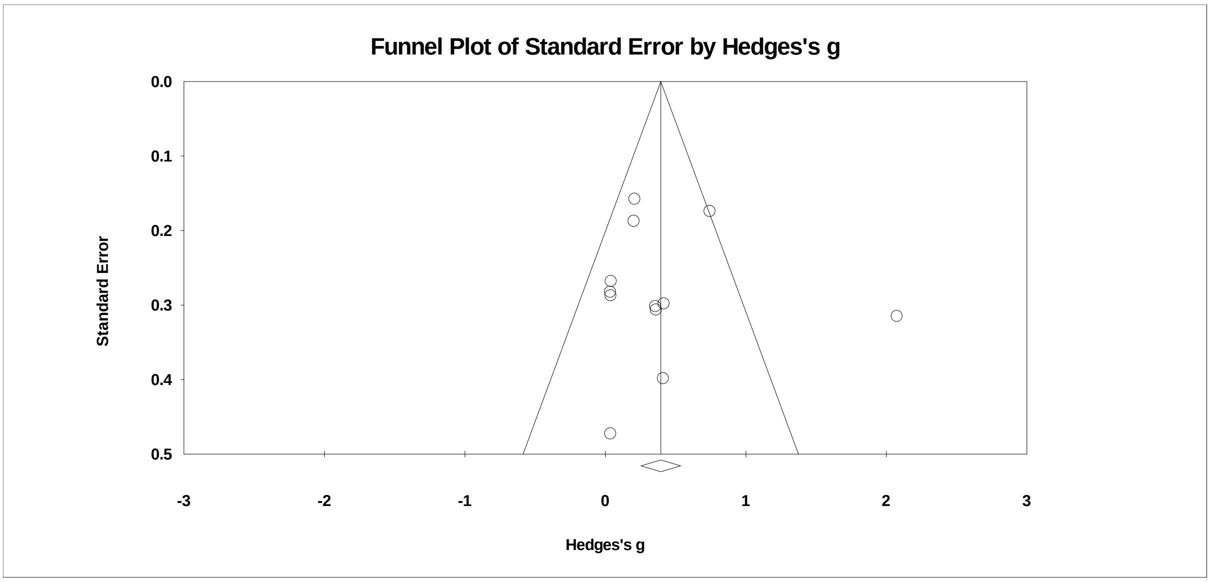

Figure S2. Subgroup Analysis on overall attention

1. Intervention Format

Group by Intervention format	Study name	Hedges's g	Standard error	Variance	Lower limit	Upper limit	Z-Value	p-Value
Group	Doshi et al., 2021a	0.417	0.298	0.089	-0.167	1.002	1.399	0.162
Group	Doshi et al., 2021b	0.362	0.306	0.094	-0.238	0.963	1.182	0.237
Group	Vidovich et al., 2015	0.210	0.158	0.025	-0.100	0.519	1.328	0.184
Group	Pooled	0.273	0.127	0.016	0.025	0.522	2.153	0.031
Group	Prediction Interval							
individual	Duff et al., 2022	0.204	0.187	0.035	-0.163	0.571	1.088	0.277
individual	Fiatarone Singh et al., 2014a	0.036	0.283	0.080	-0.518	0.590	0.129	0.898
individual	Fiatarone Singh et al., 2014b	0.041	0.268	0.072	-0.485	0.566	0.151	0.880
individual	Finn et al., 2011	0.038	0.473	0.224	-0.889	0.964	0.079	0.937
individual	Finn et al., 2015	0.412	0.399	0.159	-0.369	1.194	1.035	0.301
individual	Pantoni et al., 2017	0.358	0.302	0.091	-0.234	0.949	1.185	0.236
individual	Torpil et al., 2021	2.077	0.315	0.099	1.459	2.695	6.592	0.000
individual	Li et al., 2019	0.744	0.174	0.030	0.403	1.085	4.271	0.000
individual	Barnes et al., 2009	0.039	0.287	0.083	-0.524	0.602	0.135	0.892
individual	Pooled	0.444	0.201	0.040	0.050	0.838	2.209	0.027
individual	Prediction Interval	0.444			-0.883	1.772		
Overall	Prediction Interval	0.322			-0.634	1.278		

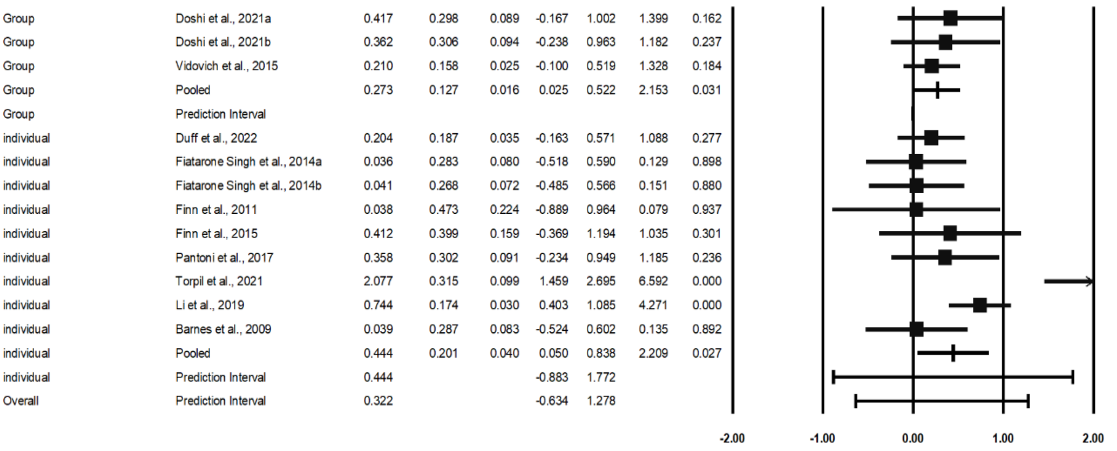

2. Length of Training (mins)

Group by Length of training (mins)	Study name	Hedges's g	Standard error	Variance	Lower limit	Upper limit	Z-Value	p-Value	Relative weight
<60	Duff et al., 2022	0.204	0.187	0.035	-0.163	0.571	1.088	0.277	34.36
<60	Torpil et al., 2021	2.077	0.315	0.099	1.459	2.695	6.592	0.000	30.99
<60	Li et al., 2019	0.744	0.174	0.030	0.403	1.085	4.271	0.000	34.64
<60	Pooled	0.972	0.450	0.203	0.089	1.854	2.158	0.031	
<60	Prediction Interval	0.972			-10.084	12.027			
≥60	Doshi et al., 2021a	0.417	0.298	0.089	-0.167	1.002	1.399	0.162	9.69
≥60	Doshi et al., 2021b	0.362	0.306	0.094	-0.238	0.963	1.182	0.237	9.18
≥60	Fiatarone Singh et al., 2014a	0.036	0.283	0.080	-0.518	0.590	0.129	0.898	10.79
≥60	Fiatarone Singh et al., 2014b	0.041	0.268	0.072	-0.485	0.566	0.151	0.880	11.98
≥60	Finn et al., 2011	0.038	0.473	0.224	-0.889	0.964	0.079	0.937	3.86
≥60	Pantoni et al., 2017	0.358	0.302	0.091	-0.234	0.949	1.185	0.236	9.46
≥60	Vidovich et al., 2015	0.210	0.158	0.025	-0.100	0.519	1.328	0.184	34.62
≥60	Barnes et al., 2009	0.039	0.287	0.083	-0.524	0.602	0.135	0.892	10.43
≥60	Pooled	0.194	0.093	0.009	0.012	0.376	2.093	0.036	
≥60	Prediction Interval								
undisclosed	Finn et al., 2015	0.412	0.399	0.159	-0.369	1.194	1.035	0.301	100.00
undisclosed	Pooled	0.412	0.399	0.159	-0.369	1.194	1.035	0.301	
undisclosed	Prediction Interval								
Overall	Pooled	0.235	0.089	0.008	0.061	0.409	2.654	0.008	
Overall	Prediction Interval	0.235			-0.711	1.182			

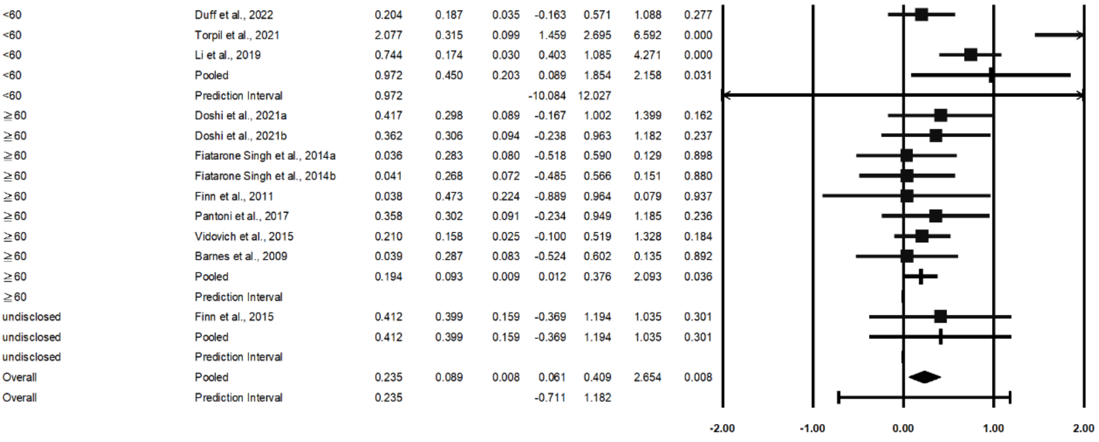

3. Weekly Training sessions

Group by Weekly training sessions	Study name	Hedges's g	Standard error	Variance	Lower limit	Upper limit	Z-Value	p-Value	Relative weight
<3	Doshi et al., 2021a	0.417	0.298	0.089	-0.167	1.002	1.399	0.162	16.69
<3	Doshi et al., 2021b	0.362	0.306	0.094	-0.238	0.963	1.182	0.237	16.51
<3	Finn et al., 2015	0.412	0.399	0.159	-0.369	1.194	1.035	0.301	14.46
<3	Pantoni et al., 2017	0.358	0.302	0.091	-0.234	0.949	1.185	0.236	16.61
<3	Torpil et al., 2021	2.077	0.315	0.099	1.459	2.695	6.592	0.000	16.32
<3	Vidovich et al., 2015	0.210	0.158	0.025	-0.100	0.519	1.328	0.184	19.43
<3	Pooled	0.628	0.275	0.076	0.089	1.167	2.282	0.022	
<3	Prediction Interval	0.628			-1.215	2.471			
≥3	Duff et al., 2022	0.204	0.187	0.035	-0.163	0.571	1.088	0.277	23.92
≥3	Fiatarone Singh et al., 2014a	0.036	0.283	0.080	-0.518	0.590	0.129	0.898	16.80
≥3	Fiatarone Singh et al., 2014b	0.041	0.268	0.072	-0.485	0.566	0.151	0.880	17.73
≥3	Li et al., 2019	0.744	0.174	0.030	0.403	1.085	4.271	0.000	25.05
≥3	Barnes et al., 2009	0.039	0.287	0.083	-0.524	0.602	0.135	0.892	16.50
≥3	Pooled	0.255	0.159	0.025	-0.057	0.566	1.604	0.109	
≥3	Prediction Interval	0.255			-0.730	1.239			
undisclosed	Finn et al., 2011	0.038	0.473	0.224	-0.889	0.964	0.079	0.937	100.00
undisclosed	Pooled	0.038	0.473	0.224	-0.889	0.964	0.079	0.937	
undisclosed	Prediction Interval								
Overall	Pooled	0.324	0.132	0.017	0.065	0.583	2.452	0.014	
Overall	Prediction Interval	0.324			-0.648	1.295			

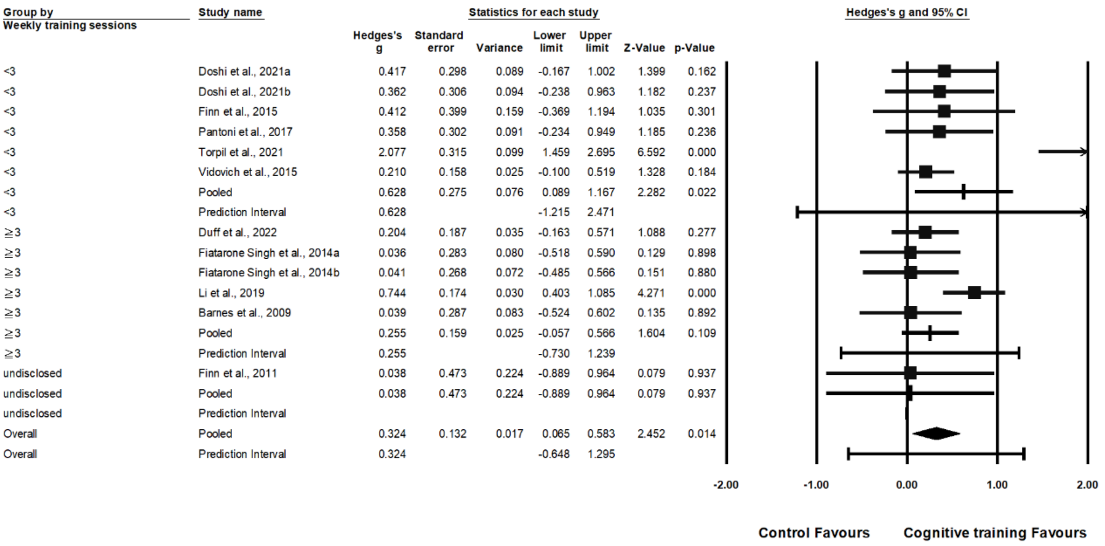

4. Total Week

Group by Total weeks	Study name	Hedges's g	Standard error	Variance	Lower limit	Upper limit	Z-Value	p-Value	Relative weight
<8	Finn et al., 2015	0.412	0.399	0.159	-0.369	1.194	1.035	0.301	10.75
<8	Vidovich et al., 2015	0.210	0.158	0.025	-0.100	0.519	1.328	0.184	68.59
<8	Barnes et al., 2009	0.039	0.287	0.083	-0.524	0.602	0.135	0.892	20.67
<8	Pooled	0.196	0.131	0.017	-0.060	0.452	1.500	0.134	
<8	Prediction Interval								
≥8	Doshi et al., 2021a	0.417	0.298	0.089	-0.167	1.002	1.399	0.162	11.89
≥8	Doshi et al., 2021b	0.362	0.306	0.094	-0.238	0.963	1.182	0.237	11.72
≥8	Duff et al., 2022	0.204	0.187	0.035	-0.163	0.571	1.088	0.277	14.05
≥8	Fiatarone Singh et al., 2014a	0.036	0.283	0.080	-0.518	0.590	0.129	0.898	12.20
≥8	Fiatarone Singh et al., 2014b	0.041	0.268	0.072	-0.485	0.566	0.151	0.880	12.50
≥8	Pantoni et al., 2017	0.358	0.302	0.091	-0.234	0.949	1.185	0.236	11.81
≥8	Torpil et al., 2021	2.077	0.315	0.099	1.459	2.695	6.592	0.000	11.54
≥8	Li et al., 2019	0.744	0.174	0.030	0.403	1.085	4.271	0.000	14.29
≥8	Pooled	0.519	0.204	0.041	0.119	0.918	2.546	0.011	
≥8	Prediction Interval	0.519			-0.825	1.862			
undisclosed	Finn et al., 2011	0.038	0.473	0.224	-0.889	0.964	0.079	0.937	100.00
undisclosed	Pooled	0.038	0.473	0.224	-0.889	0.964	0.079	0.937	
undisclosed	Prediction Interval								
Overall	Pooled	0.277	0.107	0.011	0.067	0.487	2.587	0.010	
Overall	Prediction Interval	0.277			-0.679	1.233			

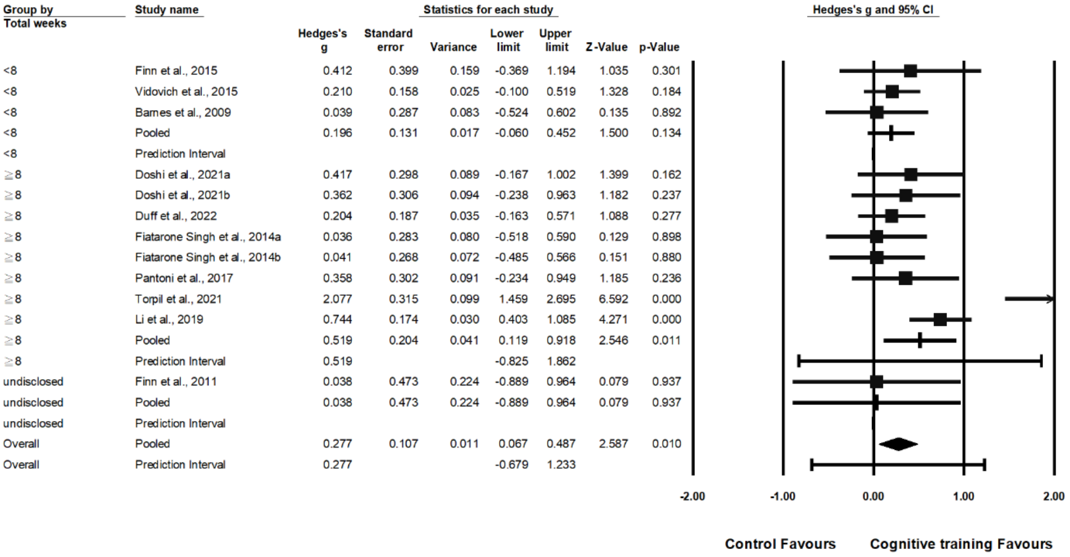

5. Total Session (session)

Group by Total session (session)	Study name	Hedges's g	Standard error	Variance	Lower limit	Upper limit	Z-Value	p-Value	Relative weight
<24	Doshi et al., 2021a	0.417	0.298	0.089	-0.167	1.002	1.399	0.162	14.17
<24	Doshi et al., 2021b	0.362	0.306	0.094	-0.238	0.963	1.182	0.237	13.42
<24	Finn et al., 2015	0.412	0.399	0.159	-0.369	1.194	1.035	0.301	7.93
<24	Pantoni et al., 2017	0.358	0.302	0.091	-0.234	0.949	1.185	0.236	13.83
<24	Vidovich et al., 2015	0.210	0.158	0.025	-0.100	0.519	1.328	0.184	50.64
<24	Pooled	0.296	0.112	0.013	0.076	0.516	2.637	0.008	
<24	Prediction Interval								
≥24	Duff et al., 2022	0.204	0.187	0.035	-0.163	0.571	1.088	0.277	16.02
≥24	Fiatarone Singh et al., 2014a	0.036	0.283	0.080	-0.518	0.590	0.129	0.898	14.36
≥24	Fiatarone Singh et al., 2014b	0.041	0.268	0.072	-0.485	0.566	0.151	0.880	14.63
≥24	Finn et al., 2011	0.038	0.473	0.224	-0.889	0.964	0.079	0.937	10.77
≥24	Torpil et al., 2021	2.077	0.315	0.099	1.459	2.695	6.592	0.000	13.74
≥24	Li et al., 2019	0.744	0.174	0.030	0.403	1.085	4.271	0.000	16.22
≥24	Barnes et al., 2009	0.039	0.287	0.083	-0.524	0.602	0.135	0.892	14.27
≥24	Pooled	0.459	0.249	0.062	-0.028	0.947	1.847	0.065	
≥24	Prediction Interval	0.459			-1.193	2.112			
Overall	Pooled	0.324	0.102	0.010	0.123	0.524	3.163	0.002	
Overall	Prediction Interval	0.324			-0.630	1.277			

Statistics for each study

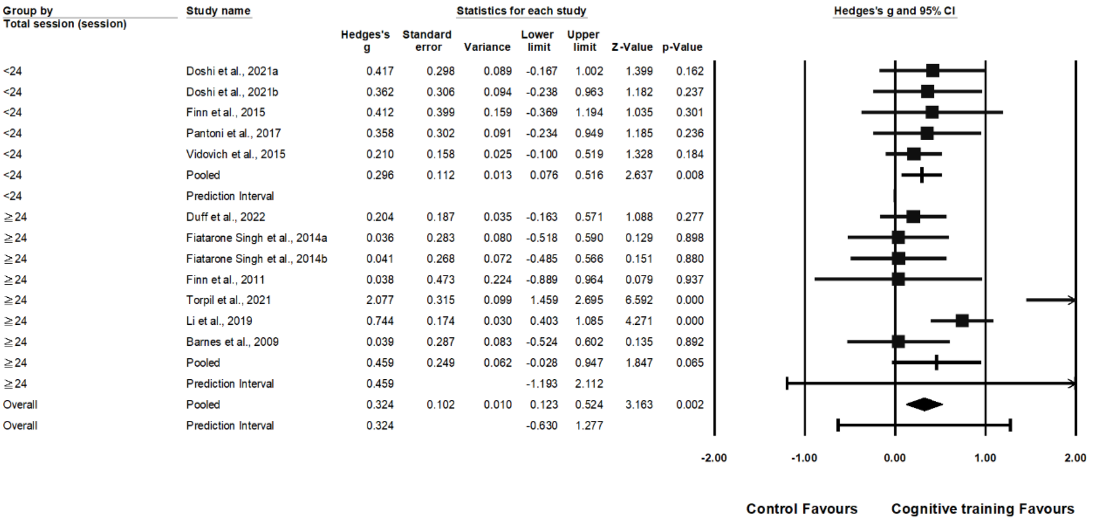

Table S5. Subgroup Analysis and Meta regression of overall attention

Variables	No. of Studies	Hedge's g (95% CI)	Null Hypothesis Test (2 tailed)		Homogeneity Test	
			Z	*p*	Q-value	*p*
**Overall attention**						
Intervention Format						
Individual	9	0.444 (0.050, 0.838)	2.209	0.027	0.516	0.473
Group	3	0.273 (0.025, 0.522)	2.513	0.031		
Overall	12	0.322 (0.112, 0.532)	3.000	0.003		
Length of training (mins)						
<60	3	0.972 (0.089, 1.854)	2.158	0.031	3.067	0.216
≥60	8	0.194 (0.012, 0.376)	2.093	0.036		
Undisclosed	1	0.412 (-0.369, 1.194)	1.035	0.301		
Overall	12	0.235 (0.061, 0.409)	2.654	0.008		
Weekly training sessions						
<3	6	0.628 (0.089, 1.167)	2.282	0.022	1.777	0.411
>3	5	0.255 (-0.057, 0.566)	1.604	0.109		
Undisclosed	1	0.038 (-0.889, 0.964)	0.079	0.937		
Overall	12	0.324 (0.065, 0.583)	2.452	0.014		
Total week						
<8	3	0.196 (-0.060, 0.452)	1.500	0.134	2.046	0.359
>8	8	0.519 (0.119, 0.918)	2.546	0.011		
Undisclosed	1	0.038 (-0.889, 0.964)	0.079	0.937		
Overall	12	0.277 (0.067, 0.487)	2.587	0.010		
Total session (session)						
<24	5	0.296 (0.076, 0.516)	2.637	0.008	0.358	0.549
>24	7	0.459 (-0.028, 0.947)	1.847	0.065		
Overall	12	0.324 (0.123, 0.524)	3.163	0.002		

Variables (Meta-regression of overall attention)	Studies	β_0	β_1	95% CI	Z	*p*
Mean of Age	12	-0.0097	0.0468	-0.1015 to 0.0821	-0.21	0.8359
